# Gastric perforation mimicking ST-segment elevation myocardial infarction

**DOI:** 10.1136/bcr-2020-237470

**Published:** 2021-03-09

**Authors:** Ryan Enast Intan, Fani Suslina Hasibuan, Parama Gandi, Firas F Alkaff

**Affiliations:** 1 Faculty of Medicine Universitas Airlangga, Surabaya, Indonesia; 2 Department of Cardiology and Vascular Medicine, Dr. R. Koesma General Hospital, Tuban, Indonesia; 3 Department of Cardiology and Vascular Medicine, Faculty of Medicine Universitas Airlangga – Dr. Soetomo General Academic Hospital, Surabaya, Indonesia; 4 Department of Internal Medicine, University Medical Center Groningen, Groningen, The Netherlands; 5 Department of Anatomy, Histology, and Pharmacology, Faculty of Medicine Universitas Airlangga, Surabaya, Indonesia

**Keywords:** ischaemic heart disease, gastroenterology, emergency medicine

## Abstract

ST-elevation myocardial infarction (STEMI) is one of the medical emergencies in cardiology with high morbidity and mortality rate which requires rapid response. In elderly patients, its presenting symptoms may be atypical which may cause the diagnosis of MI to be delayed or missed. Therefore, ST-segment elevation on ECG has become the main instrument for initial diagnosis. However, there are a variety of conditions mimicking the ECG changes of STEMI. We report a case of 70-year-old patient with acute peritonitis and pneumoperitoneum secondary to gastric perforation with dynamic ECG changes mimicking anteroseptal STEMI. After the surgery, the ECG dynamically reverted to normal. He was then discharged after 4 days without any remaining symptoms. Misinterpretation of ECG findings may lead to unnecessary aggressive intervention, costly management strategies and delay in appropriate treatment.

## Background

ST-elevation myocardial infarction (STEMI) is one of the medical emergencies in cardiology with high morbidity and mortality rate which requires rapid response. Its initial diagnosis is based on symptoms consistent with MI and signs from the ECG.^1^ In elderly patients, the presenting symptoms may be atypical (eg, shortness of breath, nausea, vomiting, fatigue, palpitations or syncope) which may cause the diagnosis of MI to be delayed or missed.^2^ Therefore, ST-segment elevation on ECG has become the main instrument for the initial diagnosis.

However, other than as a pathognomonic sign of MI, ST-segment elevation could present in variety of cardiovascular and pulmonary conditions.^3^ Moreover, it could also present in variety of abdominal conditions.^4–7^ Nonetheless, report on gastric perforation that present with ST-segment elevation was still scarce to this date. In this report, we present a case of an elderly patient with acute peritonitis and pneumoperitoneum secondary to gastric perforation with dynamic ECG changes mimicking anteroseptal STEMI.

## Case presentation

A 70 years-old Indonesian man presented at the emergency room (ER) with epigastric discomfort that was felt since one month prior and worsened in the last five days followed by nausea and vomiting. Twelve hours prior to his admission to the ER, the epigastric pain became very intense. The patient described the pain as sharp, constant and non-radiating pain. Four hours later, the pain became dull and spread to the whole abdomen, followed with additional symptoms of fever, lethargy, abdominal fullness, constipation and inability to pass wind. The patient did not have any chest pain, palpitation or shortness of breath and no modifiable risk factor for cardiovascular disease was found. The patient had a history of surgery due to gastric perforation 2 years ago and was later diagnosed with gastric ulcer due to habitual self-medication with nonsteroidal anti-inflammatory drugs and frequent consumption of spicy food even after the surgery.

## Investigations

**Figure 1:**
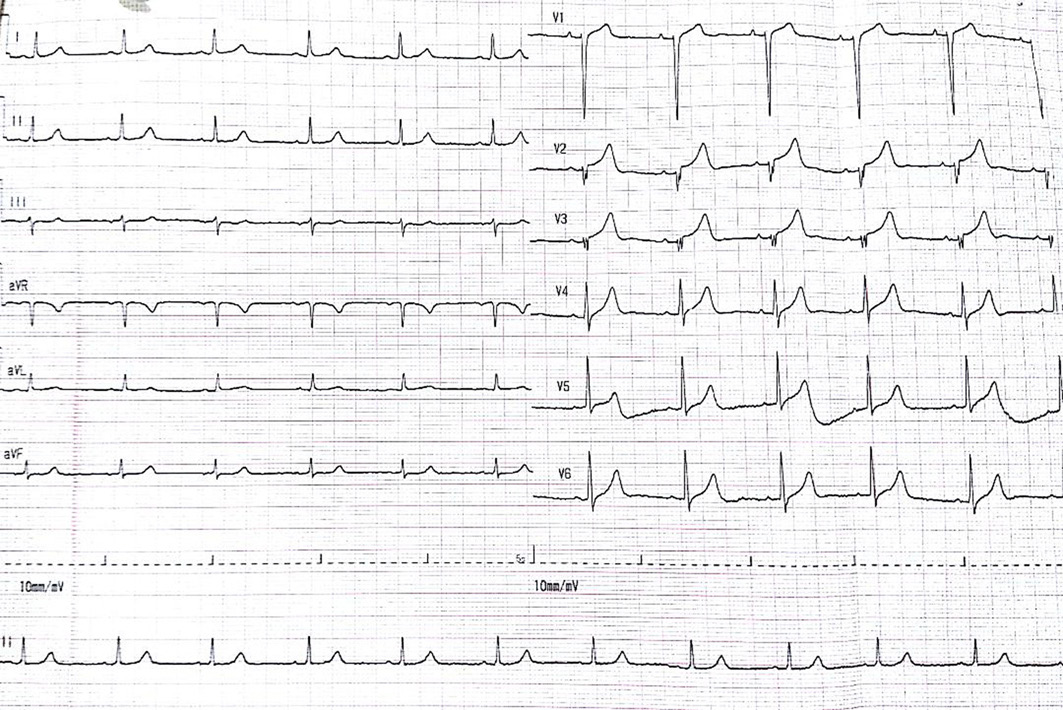
ECG evaluation on admission to emergency room showed ST-segment elevation in precordial lead (1 mm in V1, 2 mm in V2 and V3) suggesting anteroseptal myocardial infarction. aVF, augmented Vector Foot; aVL, augmented Vector Left; aVR, augmented Vector Right.

On physical examination, the patient was alert with a pulse rate of 68 beats per minute, respiratory rate of 26 times per minute, blood pressure of 110/60 mm Hg and temperature of 37.8°C. Cardiac and lung evaluation was within normal limit. The abdomen was distended with positive abdominal guarding in all quadrant, reduced bowel sounds and absent liver dullness. Surprisingly, ECG evaluation showed ST elevation in precordial lead mimicking an anteroseptal STEMI (figure 1).

**Figure 2:**
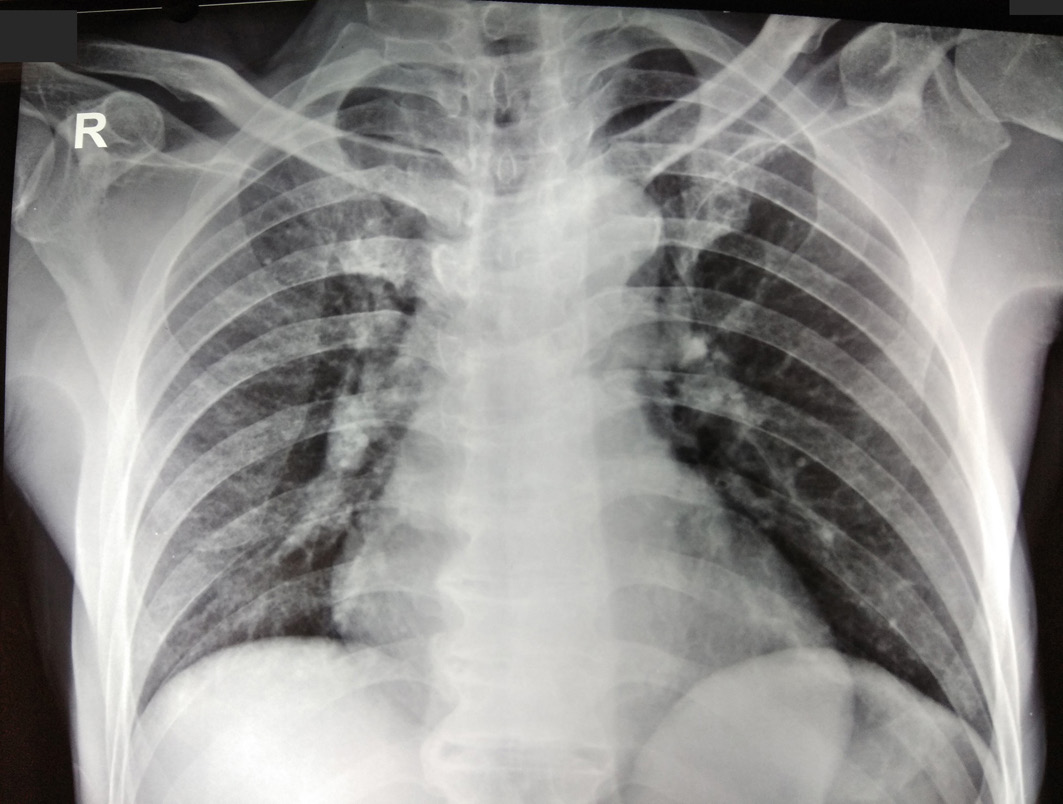
Chest X-ray anteroposterior view showed no abnormalities with cardiothoracic ratio of 47%.

**Figure 3:**
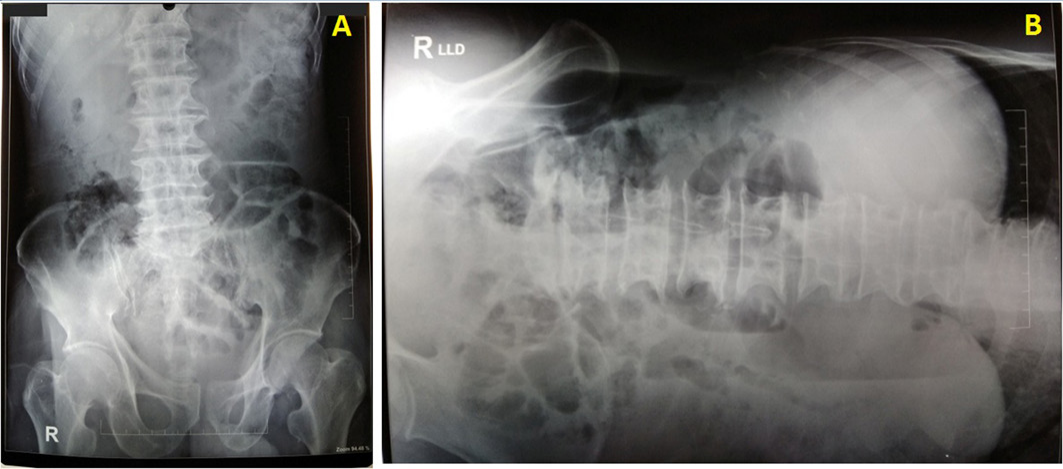
Abdominal X-ray evaluation (A) at supine position showed positive Rigler sign. (B) At left lateral decubitus position showed free air in peritoneal space.

Further examination was conducted to confirm the ECG findings. Chest X-ray showed no abnormal findings with cardiothoracic ratio of 47% (figure 2). Cardiac biomarker was within the normal limit (creatine kinase-myocardial band titre was 11 ng/mL). Due to laboratory limitation, troponin level could not be evaluated. Two-dimensional (2D) echocardiogram showed normal left ventricular function with an ejection fraction of 62%, normal heart chamber and valves without any regional wall motion abnormalities, and no sign of pericardial effusion. Abdominal X-ray showed positive Rigler sign (figure 3A) and free air in peritoneal space (figure 3B). Haematological evaluation showed leucocytosis (15.4 x 10∧9/L) while serum electrolytes, renal function test and liver function test were within normal limit.

## Differential diagnosis

STEMI should always be a differential diagnosis in patient presenting with ST-segment elevation. In a case with abdominal signs and symptoms involvement, perforation in the gastrointestinal tract should also be included as differential diagnosis.

## Treatment

The patient was consulted to the surgeon and underwent emergency laparotomy exploration. Gastric perforation was confirmed as the underlying cause in this patient.

## Outcome and follow-up

**Figure 4:**
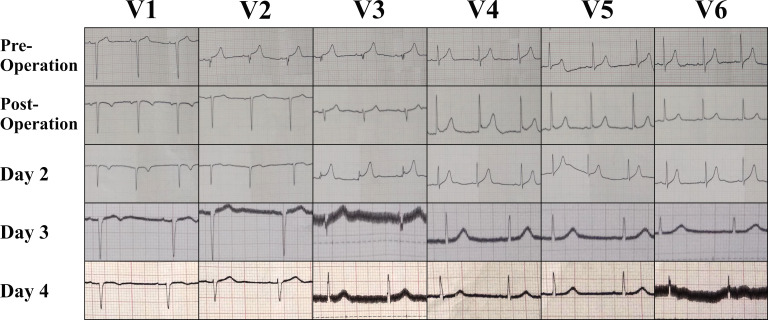
Serial ECG evaluation in precordial lead during the hospitalisation showed dynamic ST-segment changes.

After the surgery, the ECG dynamically reverted to normal (figure 4). The patient was then discharged after 4 days without any remaining symptoms.

## Discussion

ECG is a tool widely used for screening, diagnosis and management of cardiac diseases in the ER due to its availability, simplicity, noninvasiveness, less expensiveness and easy interpretation.^8^

Although ST elevation on ECG is a common finding in STEMI, it is important to note that ST elevation is not a pathognomonic sign as there are other conditions mimicking the ECG changes.^3–7^ In the literature, there have been only two reported cases of gastric perforation mimicking STEMI (table 1).^9 10^

**Table 1:** Reported cases of gastric perforation mimicking STEMI

Case	Age/sex	Chief complaints	ST-segment elevation leads	Cardiac marker	Angiography	TTE findings
Hoang *et al*^9^	70/M	Chest pain and dyspnoea	No data	No data	Minimal coronary artery disease	No data
Vutthirkraivit^10^	78/M	Epigastric pain	Anterior	Not elevated (troponin <0.01 ng/mL)	No significant stenosis	Normal wall motion with an ejection fraction of 74%
Current case	70/M	Epigastric pain	Anteroseptal	Not elevated (CK-MB 11 ng/mL)	Not performed	Normal LV wall motion with an ejection fraction of 62%

CK-MB, creatine kinase-myocardial band; LV, left ventricular; M, male; STEMI, ST-elevation myocardial infarction; TTE, transthoracic echocardiogram.

In accordance with previous cases, our case was occurred in elderly male patient. Angiography evaluation from both cases did not show significant coronary stenosis. Another report of patients with gastrointestinal distention mimicking STEMI pattern on ECG showed that from seven cases, angiography was done in four of those cases and three out of those four cases revealed normal coronary arteries.^5^

Latest guideline recommends initiating the reperfusion therapy as soon as possible in patients with a clinical suspicion of MI and ST-segment elevation to lower the rates of mortality. The following criteria to define ST-segment elevation as STEMI are: in men aged more than 40 years old, include at least two contiguous leads with ST-segment elevation of ≥2 mm in V2–3 and/or ≥1 mm in the other leads in the absence of left ventricular hypertrophy or left bundle branch block.^1^ However, the diagnosis is more challenging in elderly patients due to the atypical symptoms. There have been reports of misdiagnosis in elderly patients where cardiac catheterisation was done but revealed no evidence of obstructive coronary disease.^7 8^ In this case, the patient presented with atypical symptoms (nausea and vomiting) and ST-segment elevation (1 mm in V1, 2 mm in V2 and V3). However, other evaluations did not lead to the diagnosis of STEMI (no chest pain, no elevated cardiac enzyme, and no wall motion abnormalities or abnormal LV function in 2D echocardiogram). On the contrary, a pathognomonic finding for pneumoperitoneum was found in abdominal X-ray (free air in peritoneal space and positive Rigler sign). It is proven that peritonitis and pneumoperitoneum secondary to gastric perforation as the cause of ST segment elevation in our patient since the ECG dynamically reverted back to normal after the surgery was done.

Herath *et al* proposed that ECG changes in abdominal conditions might be due to the irritative or compressive effect to the heart or change of the heart position secondary to abdominal distention; elevated vagal tone secondary to visceral-cardiac reflex; stress-induced cardiomyopathy; or variant angina.^11^ Since the echocardiographic evaluation showed no abnormal finding and there was no resting chest pain, stress-induced cardiomyopathy and variant angina were less likely to be the cause of ECG changes in our patient. The elevated vagal tone secondary to visceral-cardiac reflex was also less likely to be occurred in our patient because the heart rate during the admission was faster than on the fourth day. Thus, the most possible mechanism of ST-segment elevation in our patient was because of the change of the heart position secondary to compressive effect of abdominal distention.

**Learning points**

- Although the presenting symptoms of myocardial infarction in elderly may be atypical and thus rely more on ECG, it should be noted that there are other conditions that could mimic ST-elevation myocardial infarction (STEMI).
- Other than as a sign for myocardial infarction, ST-segment elevation could present in variety of cardiovascular, pulmonary and abdominal conditions, including gastric perforation.
- Understanding conditions that present as STEMI is important, as misinterpretation of ECG findings may lead to unnecessary aggressive intervention, costly management strategies and delay in appropriate treatment.
